# *In Silico* Studies of Small Molecule Interactions with Enzymes Reveal Aspects of Catalytic Function

**DOI:** 10.3390/catal7070212

**Published:** 2017-07-14

**Authors:** Rajni Verma, Katie Mitchell-Koch

**Affiliations:** Department of Chemistry, McKinley Hall, Wichita State University, 1845 Fairmount, Wichita, KS 67260-0051, USA

**Keywords:** molecular dynamics simulation, catalytic activity, protein conformational dynamics, ligand interactions, cofactor dynamics, substrate access channel, solvent interactions, hydration dynamics, enzyme-substrate complex, allosteric regulation

## Abstract

Small molecules, such as solvent, substrate, and cofactor molecules, are key players in enzyme catalysis. Computational methods are powerful tools for exploring the dynamics and thermodynamics of these small molecules as they participate in or contribute to enzymatic processes. In-depth knowledge of how small molecule interactions and dynamics influence protein conformational dynamics and function is critical for progress in the field of enzyme catalysis. Although numerous computational studies have focused on enzyme–substrate complexes to gain insight into catalytic mechanisms, transition states and reaction rates, the dynamics of solvents, substrates, and cofactors are generally less well studied. Also, solvent dynamics within the biomolecular solvation layer play an important part in enzyme catalysis, but a full understanding of its role is hampered by its complexity. Moreover, passive substrate transport has been identified in certain enzymes, and the underlying principles of molecular recognition are an area of active investigation. Enzymes are highly dynamic entities that undergo different conformational changes, which range from side chain rearrangement of a residue to larger-scale conformational dynamics involving domains. These events may happen nearby or far away from the catalytic site, and may occur on different time scales, yet many are related to biological and catalytic function. Computational studies, primarily molecular dynamics (MD) simulations, provide atomistic-level insight and site-specific information on small molecule interactions, and their role in conformational pre-reorganization and dynamics in enzyme catalysis. The review is focused on MD simulation studies of small molecule interactions and dynamics to characterize and comprehend protein dynamics and function in catalyzed reactions. Experimental and theoretical methods available to complement and expand insight from MD simulations are discussed briefly.

## 1. Introduction

Proteins are nature’s best catalysts to accelerate specific biochemical reactions via catalytically reactive atoms embedded within a folded structure. The inherently dynamic nature of folded structures gives rise to an ensemble of interconverting conformations (sub-states) sampled on femtosecond to minute timescales, driven by thermodynamic fluctuations [1–3]. Characterization of conformational space, i.e., the energy landscape of a folded protein, faces several difficulties. However, advanced computer simulations [4–6], theoretical modeling [3,6,7], and spectroscopic [8,9] techniques are capable of characterizing highly populated conformational sub-states that are an intrinsic feature of protein structure and function [1,2,5].

Experimental and theoretical studies have demonstrated that catalytic function is mediated by protein dynamics and interactions with small molecules such as solvents, cofactors, and substrates [10–14]. Computational investigations involving small molecule interactions with enzymes commonly target (a) protein solvation to comprehend protein stability and flexibility, (b) protein-binding pockets and active sites to understand molecular recognition, binding affinity, and catalytic mechanisms, and (c) enzyme–substrate complexes to explore conformational heterogeneity and dynamics during catalysis. Enzyme catalysis is attributed to protein–substrate interactions, with models such as lock and key [15], induced fit [16] and conformational selection [17,18] emphasizing the role of protein conformational transitions from ligand-unbound to ligand-bound equilibrium states [19–21]. The lock and key model underpins ligand binding to native protein conformation, which is mainly determined by shape complementarity [15,22]. The induced fit model supports that ligand binding triggers protein structural changes from unbound to bound conformations [16]. However, the conformational selection model, also known as the “populations shift”, endorses the intrinsic dynamics of protein structure, which can lead to constant conformational transitions from a stable and highly populated unbound-protein state to a less stable and scarcely populated bound-protein state, which can be stabilized by a ligand binding event [17,18]. The conformational selection model has emerged as an essential component of ligand binding mechanisms, and is sometimes found to be coupled with induced fit to optimize protein–ligand interactions [18,23,24]. Buried active sites in the protein lead to a keyhole-lock-key model that supports the involvement of ligand binding tunnels in enzyme activity, specificity and stereoselectivity, and the requirement of the complementarity of ligand and binding tunnel in the ligand binding process [25].

In enzyme-mediated chemical catalysis, the dynamic nature of the protein structure plays a crucial role in molecular recognition, substrate binding, catalysis, and product release [1,3,6,11,12,26–31]. Substrate binding involves the exploration of protein conformational space to find a range of reactive conformations over microsecond to millisecond time scales and longer [1,12,20,28,32]. During the course of the reaction, catalytically relevant time scales of protein dynamics and processes range from fast harmonic motions of bonds, angles and atoms, at femtosecond to nanosecond time scales, to slower concerted motions involving large regions of the protein, at microsecond to millisecond time scales and beyond. Hence, a range of techniques is required to obtain a complete picture of enzymatic catalysis [8,19,33,34]. Advanced experimental techniques such as X-ray crystallography, nuclear magnetic resonance (NMR), and electron cryomicroscopy (cryo EM) are able to provide dominant structures, various timescales of protein motions, and exchange rates, respectively [35,36]. NMR relaxation studies showed that protein collective motions are not restricted to the active site, but to a wider dynamic network [26,37]. The collective dynamics of proteins observed during catalysis are also present in substrate-free enzymes with frequencies related to the catalytic turnover rate [19,20,26]. In summary, protein dynamics connect protein structure to function, which, in turn, are influenced by the presence of small molecules, whether they are substrate, solvent, or cofactor. Computational studies have expanded our knowledge towards understanding the role of small molecules in enzyme catalysis. In particular, molecular dynamics (MD) simulations offer site-specific information about the interactions and dynamics of small molecules in the presence of proteins (bound, at the surface, or in the solvation layer) [30,38–41].

Solvent is a critical player in protein dynamics, and thus, enzyme catalysis. Rapid fluctuations in the atomic positions of proteins lead to large-scale motions, which are observed to be slaved or coupled to solvent motions in the case of some proteins [42,43]. It is relatively more challenging to experimentally investigate solvent dynamics than protein dynamics during catalysis, and this is an area where in silico methods have made significant contributions to the field. In analysis of seminal experiments, Frauenfelder et al. have explained the role of solvent dynamics in the slaving of protein processes in native and unfolded states [42]. Such slaving does not occur in all proteins, nor are all motions of a protein slaved [44]. The characterization of protein dynamics slaving to solvent dynamics is, in fact, controversial [45]. This review highlights in silico contributions to understanding solvent dynamics in enzymatic function, with a focus on how simulations complement spectroscopic observations, and a description of the different types of analyses to characterize dynamics within the biomolecular solvation shell.

Substrate binding is often considered the key event in enzyme catalysis. Numerous studies have focused on studying the effect of translational, rotational and conformational entropy upon substrate binding [46]. MD atomistic simulations can be used with free energy perturbation methods to calculate free energies [42,47]. However, these techniques do not provide a good estimate of overall entropy changes, due to the requirement of extensive sampling and convergence of potential energy. The role of solvent entropies is even more scarcely studied than substrate entropies. Hence, contributions of solvent dynamics to enzyme catalysis are often neglected in models describing enzyme dynamics and catalysis. Combined experimental and theoretical approaches are a powerful platform to measure changes in coupled water-protein motions during enzyme catalysis. Entropic effects on reaction rates and molecular associations in enzyme catalysis are not fully understood. However, combined computational and experimental approaches promise to measure the thermodynamic contributions of water molecules in protein-substrate associations, and identify changes in water-protein interactions during various steps of enzyme reactions [48–51]. Enthalpic and entropic contributions of bound-water molecules demonstrate the role of protein–solvent interactions in the rate enhancement of enzyme-catalyzed reactions [48–51].

Atomistic aspects of protein dynamics connect the relationship between protein structure and function. MD simulations emerged as a crucial methodology in structural biology to allow the investigation of the physical mechanisms underlying protein function by examining the dynamical behavior of proteins at an atomistic level [52,53]. Although advances in MD simulations are leading to a new level of knowledge of macromolecular complexes involving millions to billions of atoms [54], the vast majority of MD studies involve relatively simple proteins when exploring substrate binding, catalytic activity, and protein structure–function–dynamics relationships. However, investigation of solvent molecules and cofactors is equally important to understand protein-mediated catalysis. A number of studies have used small molecule interactions and dynamics to characterize (a) effects on overall protein structure and dynamics, and (b) site-specific effects that influence enzyme catalysis. MD simulations concentrating on protein structure- dynamics involve: (a1) protein conformational variations, transitions and dynamics, (a2) protein surface chemistry, (a3) protein–protein interactions, (a4) allosteric regulation, and (a5) protein solvation environment (solvation shell, solvent concentration, pH, temperature and ionic strength). Meanwhile, computational studies of regional or site-specific protein features have focused on: (b1) active site conformation, dynamics, variations and environment, (b2) substrate binding and product release, (b3) substrate transport, (b4) cofactor binding and release, and (b5) catalytic mechanism and transition state pathways.

**Figure 1:**
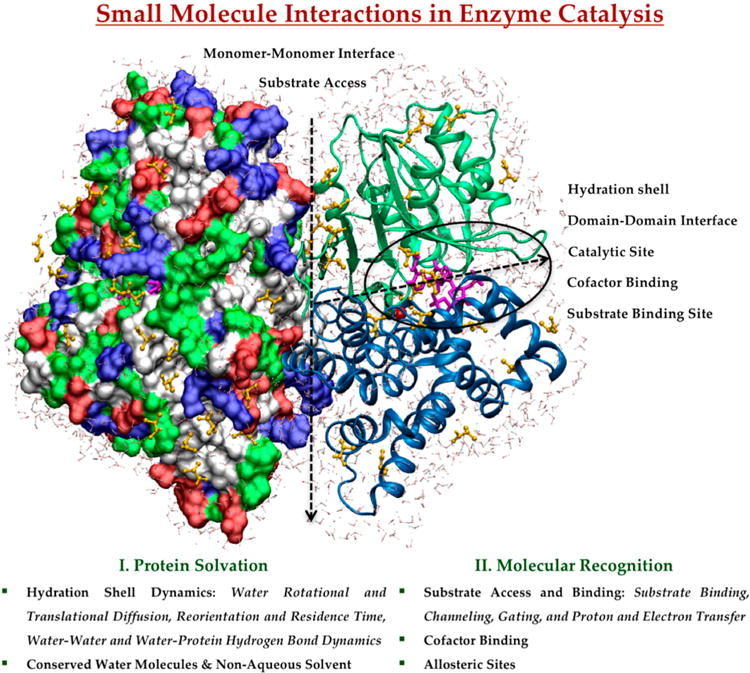
Small molecule interactions with protein are illustrated based on their involvement in (**I**) protein solvation and (**II**) molecular recognition using a model protein, YqhD aldehyde reductase (PDB ID: 1OJ7 [55]). The dotted arrows show the monomer and domain interfaces in the YqhD homodimer. The monomers are in a surface representation colored by residue type, and in a cartoon representation with the domains colored in blue and green, respectively. The encircled region includes reduced nicotinamide adenine dinucleotide phosphate (NADPH) and zinc ion cofactors, and isobutyraldehyde substrate binding in the catalytic site. Substrate and water molecules within 3.5 Å of protein represent the hydration shell and substrate binding on the protein surface.

This review provides an overview of recent progress towards understanding the role of small molecule interactions in enzyme catalysis using MD simulations as a crucial tool to characterize, comprehend and exploit natural catalysts. We will summarize selected MD studies that have addressed the effect of small molecule interactions and dynamics on protein structure, function, and dynamics. We will emphasize what kinds of information are still unclear and require further studies at molecular level. For simplicity, we have organized protein-based small molecule interactions according to their involvement in protein solvation as a solvent, and in molecular recognition of substrate and cofactor, schematically represented in Figure 1. These play roles in protein conformational dynamics and transitions, ligand binding and release, catalytic mechanisms, and allosteric regulation in the catalytic reaction.

## 2. Effect of Small Molecule Interactions on Protein Structure, Function, and Dynamics

### 2.1. Protein Solvation in Enzyme Catalysis

#### 2.1.1. Hydration Shell Dynamics

Water is an integral component of biomolecular function [56]. Being in close proximity to the protein, it plays an important role in many physiological functions and is crucial to protein folding, protein stability, molecular recognition, ligand binding or release, and catalytic activity [13,57–59]. Using the distance of a water molecule from the protein surface, water can be categorized as bulk water, hydration water, and bound water [60–62]. Bulk water molecules maintain a distance longer than the van der Waals radius from the protein surface and facilitate protein diffusion relative to other interacting molecules. Hydration water interacts closely with protein and bulk water, and contributes to protein structure, function and dynamics by forming a hydrogen-bonded water network around the protein surface. However, bound water, identified in crystallographic structures, is often involved in the internal hydration of protein, and maintains multiple hydrogen bonded contacts with amino acid residues, ligands and water molecules located in or near buried cavities and ligand binding sites [63]. Bound waters, also known as “ordered water molecules [64]”, have been observed in protein structures studied by crystallography (X-ray [65,66] and neutron [67–70] scattering) and spectroscopy (NMR [37,71], and 2D IR [72]); sometimes in combination with high throughput small-angle X-ray scattering (SAXS) studies [73–76] and MD simulations [13,77,78]. Often, bound waters remain conserved among related proteins and contribute to structural stability and plasticity by forming extensive hydrogen bonded networks; it is sometimes found to be involved in hydration water networks [61,79]. Rodríguez-Almazán et al. showed that the substitution of conservative amino acid leads to altered enzyme function due to disruption of conserved water molecules and water-mediated networks [80].

The dynamics of the hydration layer around the protein (“hydration dynamics”) have been subjected to various experimental and theoretical studies [59,61,78,81]. Protein hydration studies have focused mainly on exploring water structure and protein–water interactions with surfaces, and usually involve one or two hydration shells or solvation layers [68,76,81,82]. The heterogeneous nature of water dynamics in the hydration shell may possibly facilitate conformational transitions occurring during catalytic transformation [68,71,76]. Recently, mass spectrometry (MS) emerged as an important method to comprehend protein dynamics and conformational changes in solution using covalent labeling, hydrogen–deuterium exchange (HDX-MS) and hydroxyl radical footprinting (HRF-MS) [83–88]. HDX-MS [84] provides time resolution of the protein motions relevant to covalent modifications, allosteric regulation, conformational heterogeneity, and catalytic function by analyzing protein–protein and protein–ligand interfaces [85,86,89]. Meanwhile, HRF-MS [90] is applicable for quantitative structural mapping of proteins by providing detailed information about the solvent accessibility of individual amino acid side chains [85,86,89].

Two-dimensional infrared echo spectroscopy (2D IR) provides measurements of protein conformational fluctuations and hydrogen bond (H-bond) dynamics in the solvation shell [72,91]. MD simulations complement 2D IR data to obtain hydration shell dynamics by computing water-protein H-bond lifetimes using H-bond survival time correlation functions (TCFs). The H-bond TCF is defined as < *s*(0) · *s*(*t*) > where *s*(*t*) = 1 for an intact hydrogen bonding pair, and *s*(*t*) = 0 when the H-bond is broken. The TCF is averaged over time and all waters (for these purposes, within the solvation shell). Fitting of the H-bond existence TCFs to an exponential curve (single, multi-exponent, or stretched) is then used to extract H-bond lifetimes. Water-protein H-bond lifetimes acquired from H-bond existence TCFs provide a measure of hydration shell dynamics, while also reporting on the influence of protein structure on the dynamics of solvation layer waters. Other components of hydration shell dynamics that have been examined by MD simulations include residence times within the hydration shell, which report on time spent by a solvent molecule in the hydration layer(s), and are related to diffusion tangent to the protein surface [92]. Residence times are typically calculated from MD trajectories with a similar methodology to H-bond lifetimes, using survival probability time correlation functions. Reorientation times of water in biomolecular solvation shells have been studied extensively in a number of simulation studies [13,93–95]. Reorientation times are measured experimentally by NMR and dielectric spectroscopy, and solvation layer reorientation times are related to the local viscosity of the solvent [96]. The reorientational dynamics of individual water molecules were calculated to be moderately perturbed in the protein hydration shell, with differences originating from local surface topology and the chemical nature of protein atoms at the surface [13,97].

Atomistic MD simulations and clustering analyses have proven critical to complement experiments that characterize protein solvation, and to obtain consistent hydration site predictions [98]. The different tools and methods available to complement MD simulations have been reviewed recently [13,57,99–101]. Post processing of MD trajectories using programs such as WaterMap [102], and WATSite [103] predict the location and thermodynamic properties of hydration sites. Clustering techniques were found to be critical in order to overcome the influence of simulation lengths and starting conformations on the prediction of hydration sites and desolvation energies of protein due to the replacement of water molecules upon ligand binding [71,98]. MD simulations have been performed on a set of proteins to investigate how protein solvation is affected by environmental changes, such as an increase in the temperature [97], and by adding urea or crowding agents [62,104]. Water dynamics within the hydration shell can be analyzed through reorientation dynamics and residence times of water, local tetrahedral order in water, distribution and retardation maps of water around protein, and H-bond strength between water and protein. Water reorientation times obtained from MD simulations are found to be comparable to reorientation times measured with NMR experiments [105]. The results illustrate the high degree of dynamics and plasticity of water molecules that keep proteins solvated under various conditions [62].

In charge transfer processes, reaction rates depend on solvent or protein rearrangements [19,20,106–110]. Kinetic terahertz spectroscopy and X-ray absorption spectroscopy of stopped-flow enzyme catalysis have identified changes in coupled protein–water dynamics concomitant with the formation of the catalytically-active Michaelis complex in a membrane type 1 matrix metalloproteinase (MT1-MMP), observing retardation in the active site hydration shell during this step [10]. The dynamical influence of solvent extends beyond active site rearrangements. For instance, protein dynamics have been shown to couple to hydration dynamics [91,111], with water acting as a lubricant or driver for side chain motions and the conformational dynamics of proteins [104,112,113]. Kubarych et al. investigated site-specific coupling of protein–water dynamics using elastic network normal mode analysis [114] (NMA) [91]. The results suggested that the susceptibility of protein motion to solvent involves changes in protein surface topology accompanied by solvent rearrangement. Specially, large amplitude motions with collective and low frequency modes are susceptible to solvent slaving and found to influence catalytic function such as ligand binding and release [91]. Neutron diffraction studies show the onset of protein motion concomitant with increased translational dynamics of water [115]. A combined study of quasi-elastic neutron scattering and MD simulations showed that the translational diffusion of hydration water promotes large amplitude motions in proteins to facilitate the dynamical transitions required for biological activity [115].

It appears that protein structure influences hydration shell dynamics, which in turn are coupled to enzyme dynamics and function [13,76]. Biomolecular surfaces affect the structure and dynamics of hydration shell waters in a number of ways. The relative orientation of water molecules in biomolecular hydration shells is not random, and is most probably driven by hydrogen bonding [13,42,68]. Hydration shell dynamics have been found to be affected by protein conformational states or conformational fluctuations in some cases, especially at regions on the protein surface involved in the partial confinement of hydration water molecules [13,97]. Slow diffusion of water molecules was observed on the peptide surface by neutron scattering, NMR spectroscopy, and mutation experiments by analyzing H-bond network dynamics in hydration layers [69,97,116]. For example, Halle et al. studied the dynamical heterogeneity of protein-water interfaces using NMR relaxation experiments and MD simulations [116]. They analyzed the solvent-accessible surface area derived hydration number, correlation time distribution [117], rotational correlation time, and dynamic perturbation of solvent using MD trajectories [116]. Terahertz Raman spectra of proteins (in ultra-fast optical Kerr-effect spectroscopy) are capable of characterizing variations in solvent–protein hydrogen bonding and dynamics on a picoseconds timescale [118,119]. The picosecond dynamics of the protein–water H-bond network determined by MD simulations can be correlated with terahertz spectra of protein solution [120]. In a fascinating example of this, terahertz spectroscopy coupled with MD simulations has revealed a “hydration funnel” or gradient in hydration shell dynamics of metalloproteinase MT1-MMP in which the water dynamics surrounding substrate and protein slow down as the substrate approaches the active site [111]. The extent of water retardation in this case was found to be substrate-dependent, revealing the unique nature of small molecules in enzyme catalysis. Computational analysis of molecular dynamics in the MT1-MMP work, involving calculation of water–water H-bond survival probability TCFs, complemented measurements by terahertz spectroscopy [121]. The same investigators undertook a combined spectroscopic, kinetic, and MD investigation that provided microscopic details about the changes in protein–solvent motions upon substrate binding. Water hydration dynamics were found to change at different stages of the enzymatic reaction, while the retardation of water dynamics was connected to the formation of an enzyme–substrate complex during catalysis [10]. Further evidence for hydration dynamics providing critical attenuation for catalysis was recently provided by Han and co-workers, who studied the water molecules around a series of single-chain polymeric nanoparticles that had varying levels of catalytic activity [122]. It was found that the catalytically-active nanoparticles had a hydration retardation similar to that around a protein, whereas the catalytically-inactive nanoparticles did not have the same hydration dynamics signature.

The extent to which dynamics of the solvation layer influence enzymatic catalysis is still being determined. However, in silico studies are poised to contribute significantly to our understanding of a solvent’s role in catalysis, particularly regarding how the dynamics and dynamic fluctuations of solvent are coupled to enzyme conformational dynamics, active site rearrangements, molecular recognition and ligand binding.

#### 2.1.2. Conserved Water Molecules and Non-Aqueous Solvent

Water molecules work as a lubricant to facilitate protein dynamics [104], yet serve as glue in binding interfaces [113], leading to conclusions that protein function is critically coupled to hydration [43,57,123]. Generally, dehydration of amino acid residues or loss of the hydration layer in proteins results in loss of activity and flexibility [78], while internal hydration of proteins is correlated with its conformational space [124,125]. Water molecules located at interfacial regions and in the active site, identified in crystallographic structures, often play structural and functional roles [61]. Bound water has been observed in different crystallographic structures at specific positions, such as in polar cavities, to enhance protein stability, or in the active site, to participate in ligand binding or catalysis. When a ligand binds to a protein, bound-water molecules in the active site can be displaced by the ligand, also known as “displaceable water”, or they can remain bound to participate in water-mediated interactions between protein and ligand, in which case they are deemed “conserved water” [46,65,126]. In both conditions, bound water influences the shape and energetics of protein–ligand associations [46,126]. Water binding sites can be identified by using programs such as WaterDock [127], which combines data mining, heuristic and machine learning techniques, and WaterScore [128], which uses multivariate logistic regression analysis to predict if a water molecule can be displaced or will remain conserved in the ligand binding site. However, PyWATER [129] simply identifies conserved water molecules in proteins using a clustering method.

Conserved water molecules have been identified in the active site and substrate-binding cleft in protein kinases using MD simulations and free energy calculations [79]. In one study, crystallographic structure ensembles were used to identify clusters of conserved water molecules at positions critical for structural stabilization and peptide binding in major histocompatibility complex (MHC) class-I-peptide complexes [130]. Moreover, MD simulations of serine protease factor Xa (fXa) demonstrated that internal water clustering serves as an integral part of the protein starting structure; these are required when initializing MD simulations to achieve stable trajectories and realistic dynamics [131]. Furthermore, internal water clusters contribute to H-bond networking in the heavy chain protein of fXa and were observed at its ligand binding site, entrance to the substrate access channel, and along the protein surface. Fox et al. studied the thermodynamics of protein–ligand associations in human carbonic anhydrase II (HCAII) using calorimetric, crystallographic, site-directed mutagenesis, and computational studies [49]. Mutation-derived changes in the entropic and enthalpic contributions of bound-water molecules were determined using WaterMap [42,102]. The results demonstrate that the thermodynamics of protein–ligand association is influenced by the organization of water within hydrogen-bonded networks in the binding pocket. In another example, water structure and dynamics were studied in the active site of *Thermus thermophiles* β glycosidase by combining crystallographic, deuterium-exchange mass spectroscopic (DXMS), and MD simulation data [132]. The simulation results complemented the DXMS results and identified internal water channels extending from the protein surface to buried acid-base residues involved in the catalytic mechanism. These conserved water molecules were found to have longer residence times: on nanosecond, rather than picosecond, timescales. Syrén et al. combined site-directed mutagenesis and computational methods to demonstrate that the entropic contributions from the release of water through specific channels enhances the rate of an enzyme-catalyzed polycyclization reaction [48]. Caver [133] software was used to perform water channel analysis on snapshots obtained from MD simulations of the triterpene cyclase from *Alicyclobacillus acidocaldarius*. The role of buried and conserved water molecules has been investigated recently using all available crystal structures and conformations derived from independent MD simulations of a signaling protein Q61H K-ras in the presence and absence of selected crystallographic water molecules [63]. Buried water molecules show strong coupling with local and global protein motions, and influence sampling of protein conformational states such as active GTP-bound, intermediate GTP-bound, inactive GDP-bound, and nucleotide-free conformations. Water-mediated correlated motions involve functionally crucial regions (switch 2, P loop and helix 3) to modulate transitions between populated conformational states. High-residence water molecules were found to act as allosteric ligands to induce a population shift to the functionally distinct conformations of switch 2, which take part in effector recognition.

In spite of water’s integral role in enzyme function, it has been found that some enzymes function in non-aqueous environments, either in organic solvents or ionic liquids [134,135]. MD simulations of proteins in non-aqueous solvent give evidence for the stripping of highly dynamic and weakly-bound water molecules from the protein surface [136] and active site region, providing atomistic insight into protein solvation, stability, and catalytic efficiency [134,137]. Simulation results show that the nature of organic solvents influences the extent of stripping of water molecules from the protein surface: non-polar solvents give rise to tightly bound large water clusters on the protein surface, while polar solvents promote loosely bound small clusters of water [136]. A recent MD simulation study of *Candida antarctica* lipase B (CALB) and cytochrome *c* enzymes indicates that retaining buried waters in the presence of different non-aqueous solvents leads to faster-equilibrating MD trajectories [138]. Furthermore, retaining buried waters affects the conformational sampling of CALB in organic solvents. This work by Mitchell-Koch and co-workers also provides guidance for MD methodology in non-aqueous solvent via judicious choice of which crystallographic waters to include when initializing simulations. Meanwhile, MD simulations of γ-chymotrypsin combined with quantum mechanics (QM) methods in acetonitrile media showed decreased enzymatic activity (relative to aqueous solution) due to weakening of the hydrogen bonding environment in the active site and an increased proton transfer barrier [139]. QM methods complement semi-empirical MD methods, and are often restricted to the protein active site to study chemical reactions in terms of bond making and breaking, and to characterize molecular interactions at the electronic level. Simulations of trypsin demonstrated the effect of solvent environment on enzymatic structure, substrate binding, solvent distribution, and catalytic hydrogen bonding using both a substrate-free and a substrate-bound enzyme [140]. During simulations, acetonitrile strips off water molecules from the protein surface, and shows higher deviation from the crystal structure than hexane. However, stronger substrate binding was observed in hexane due to a strengthened H-bond network in the active site resulting from conformational changes in the binding surface. In these simulation studies of different solvents, the protein can be characterized using structural and dynamic properties such as root mean square deviation, root mean square fluctuation, radius of gyration, secondary structure, solvent accessible surface and hydrogen bonding in the substrate binding and active sites.

Since water covers protein surfaces and is bound with different level of tightness, hydration waters are observed to exchange at different rates, and can be displaced by ligand molecules from binding sites. Organic solvent molecules have been used experimentally (in multiple solvent crystal structures (MSCS) [141,142] and NMR based fragment screening [143]), and theoretically [134] as substrate mimics to interact with the protein surface and explore energetically favorable binding sites [142,144]. Simulations have been used to map such “hot spots” on a protein. Experimental validation of such work is typically provided by MSCS, in which proteins are crystallized in the presence of various organic co-solvents, and regions having co-solvent contacts across multiple solvents are identified as hot spots [141]. Hotspot mapping through co-solvent MD simulations is usually done for medicinal chemistry applications to identify allosteric sites, in the context of drug discovery [145,146]. Desolvation of protein–ligand interacting surfaces results from favorable interactions, and arises from a balance of entropic and enthalpic forces.

A better understanding of water-mediated interactions between ligand and protein also can play a crucial role in rational drug design [99]. Water molecules can either be conserved with tight binding in the active site or can be displaced by the ligand [126]. MD simulations of proteins in the presence of mixed solvents have potential to map not only the general binding site on surfaces but also identify active sites, cofactor binding sites, protein interfaces, and allosteric sites, which might be crucial to understand and modify specificity for different compounds, particularly in computational drug design applications [145,147].

### 2.2. Molecular Recognition in Enzyme Catalysis

#### 2.2.1. Substrate Access and Binding

##### Substrate Binding

Enzymes lower the activation energy of chemical reactions and bring substrates to the active site in a suitable orientation for an enzyme–substrate complex, which often involves the rearrangement of atoms in the active site. Ligand binding models propose that the binding of the substrate in the active site could be accompanied by conformational change [5,20,21]. Indeed, different conformations have been observed for apo- and holo-protein (without and with bound substrate/cofactor, respectively) in experimental and computational studies. Even the “native state” of a substrate-free protein comprises a large number of conformations that correspond to local minima in the potential energy surface of the system. Conformational flexibility of proteins is also required for substrate binding, catalytic conversion, and product release. The presence of these sub-states, and transitions between some of the sub-states, has been studied computationally and experimentally using different systems [5,8,19]. However, the relationship between enzyme flexibility and activity is still being actively investigated, and is complicated by the fact that functionally relevant protein motions occur at different length and time scales [33]. Ligand binding increases the population of sub-states with small-scale (side chain rearrangement) and large-scale (subdomain rotation and alternate loop conformation) conformational changes that promote enzyme–substrate association and catalysis. Small-scale protein motions take place on the picosecond to nanosecond timescale for the displacement of atoms, and nanosecond to microsecond timescale for loop motions. Large-scale motions involving domains require high activation energies and are observed on a milliseconds or longer timescale. Time-resolved spectroscopic approaches have proven useful to characterize protein conformational heterogeneity and dynamics in enzymatic catalysis [148]. The vibrational modes of substrates are sensitive to local environments, and can serve as reporters of changes in protein conformation, etc.; thus, vibrational spectroscopy is crucial to decipher the energy landscape relevant to catalysis, and can observe bond reactivity in the enzyme–substrate complex [148].

The relationship between protein dynamics and reaction rates is indicated by MD simulation studies in light of structural (X-ray and NMR), kinetic, and mutagenesis data. For instance, protein dynamics were found to be affected by small changes in the ligand structure during MD simulations of ternary complexes of nicotinamide adenine dinucleotide phosphate (oxidized NADP^+^ or reduced NADPH) bound dihydrofolate reductase (DHFR) with 7,8-dihydrofolate as substrate, and 5,6,7,8-tetrahydrofolate as product [149]. Conformational changes and motions in the catalytically important loop regions of DHFR ternary complexes were characterized using properties such as order parameters, relaxation time, and B-factors, which can be compared to NMR and crystallographic measurements. The Michaelis complex exhibits strong correlations in the movement of the catalytically important FG and M20 loops. These correlated motions also involve distant regions of the protein structure in the reactant complex, while they are absent in the product complex [149]. These motions facilitate an increase in the frequency of barrier crossing through transition states of the enzyme-catalyzed reaction. Further characterization of these motions in DHFR makes evident their involvement in the reorganization of the environment to facilitate hydride transfer by decreasing the distance between donor and acceptor, orienting substrate and cofactor, and providing a favorable electrostatic environment [108]. MD simulations of substrate-free and -bound cyclophilin A (CypA) also give evidence that the motions of active site residues in a substrate-bound complex are inherent in the substrate-free enzyme [150]. Principle component analysis (PCA) was used to characterize the molecular recognition mechanism of CypA upon substrate binding by revealing similarities between substrate-free and substrate-bound ensembles of enzyme conformations. In this same work, stabilization of the loop region (residues 66–96 of CypA) upon substrate binding suggests the importance of loop motion in Michaelis complex formation. A theoretical investigation of CypA used the dynamic reaction path (DRP) method, which characterizes the network of vibrations that promote catalysis and rate enhancement, by adding kinetic energy to protein vibrational modes corresponding to conformational fluctuations in the network. The DRP analysis indicated contributions from many of the loop region residues to the top three vibrational modes of CypA [151]. In the Michaelis complex of CypA, changes in substrate configuration during catalysis induce structural and dynamical changes in the protein, which are observed beyond the active site and result in a narrower sampling of conformational space [150]. Experimental studies demonstrate the influence of inter-domain movement on catalytic rate by modulating the hydrogen tunneling distance in a horse liver alcohol dehydrogenase [152]. Protein–substrate interactions were found to modify the density of states, illustrating the influence of substrate binding on rate-determining protein dynamics. Different experimental and computational studies investigated free energy changes upon substrate binding; however, the reliability of their results depends on statistical accuracy and related theoretical analysis [5,20,148].

Applications of MD simulations along with combined quantum mechanics and molecular mechanics (QM/MM) [153,154] have been a crucial approach to overcome the limitations of both methods to explore substrate binding and dynamics, product distribution, and mechanistic insight into structural transitions along reaction pathways [155,156]. Chakravorty et al. used MD and QM/MM methods separately and also with potential of mean force (PMF) [157] to investigate the role of substrate dynamics in reactions catalyzed by farnesyl transferaes (FTase) and aromatic prenyltransferase (APTase), NphB [158]. The crystal structure of the FTase–substrate complex exhibits a gap of ~7.5 Å filled by solvent molecules between the substrate and zinc-bound peptide in the active site [159]. PMF simulations were performed to evaluate the free energy for a conformational change that is required to bridge the reactive centers [157]. Simulation results provide insight into the transition state in the FTase catalyzed reaction and explain the role of conformational changes to overcome the gap between substrate and zinc-bound cysteine by lowering the activation energy. NphB simulation results explained the importance of intermediate stabilization during the reaction followed by a water-mediated proton transfer, which leads to the formation, stabilization, and release of product from the active site.

##### Substrate Channeling

Beyond the active site, some enzymes clearly have regions with a high affinity for substrates, such as substrate access channels that facilitate the substrate reaching buried active sites within the protein core [160,161]. Questions of interest with respect to substrate directing or channeling in enzymes include conformational changes accompanying substrate channeling that may influence both substrate and protein dynamics [161]; the presence of gating mechanisms in substrate access [162]; and the structural components that promote substrate channeling, and affinity or molecular recognition [160,162–165]. Understanding substrate/product channeling is critical to tune or alter substrate specificity, enzyme function, and catalytic rates in protein engineering and drug design. MD simulations of enzymes with substrate have provided valuable insight into catalytic function with regards to substrate transport and affinity. For example, Cundari and co-workers simulated carbon dioxide (CO_2_) in the presence of phosphoenolpyruvate carboxykinase (PEPCK) protein, identifying tunnels by which CO_2_ exits and enters its binding site, and amino acids that increase the affinity of the protein for substrate [166]. In the case of PEPCK, experiments have shown that a single mutation can reduce the CO_2_ affinity 3.5 times [167], while other point mutations enhance CO_2_ binding [168]. Meanwhile, many mutations were found to have little to no effect on CO_2_ binding capability [168–170]. The effect of enzyme mutations on CO_2_ migration in the PEPCK enzyme demonstrate the promise of substrate–enzyme MD simulations to uncover structural features that influence substrate transport and selectivity, with an eye toward rational protein engineering. Energetically favorable CO_2_ binding sites were located on PEPCK using the Q-SiteFinder [171] program and used as the starting point for simulations, in order to understand the diffusion of CO_2_ into the active site interior. In another interesting study, Blumberger and co-workers uncovered a substrate access tunnel through MD simulations of carbon monoxide (CO) dehydrogenase–acetyl-CoA synthase, and were able to describe CO_2_ and CO transport in the enzyme, revealing the role of protein structure hydrogen-bonding and conformational dynamics in the process [172]. Long MD trajectories were used to calculate a probability density map for ligand distribution within the protein to define different binding states. Rate constants for the ligand transitions were calculated using non-equilibrium, constant pulling force simulations. Density functional theory (DFT) calculations on the active site were used to gain further insight into ligand binding kinetics. These two cases suggest a dynamic nature to the substrate channels that facilitate ligand diffusion into the protein interior. The transition of a ligand from the substrate access channel to the active site can be a rate limiting step, which is controlled by the dynamics of a few residues involved in the opening and closing of the channel [164]. MD simulations with PMF calculations revealed atomistic details regarding the kinetics and dynamics of ammonia transfer in carbamoyl phosphate synthetase (CPS) from the glutamine hydrolysis site through a 60 Å long tunnel comprised of hydrophobic and hydrophilic passages [173,174]. Mutagenesis studies indicate that the rate-determining step in ammonia transfer is located at the interaction of C232-A252-A314, which forms a narrow gate, while mutations at these sites inhibit ammonia migration [173]. Carbamate transport through a 40 Å intramolecular tunnel in the large subunit of CPS has been studied using MD simulations and site-directed mutagenesis studies [175]. Simulations revealed that conformational changes induce opening of the tunnel, along with prevention of charge–charge repulsion from three glutamate residues during intermediate passage through this segment. Mutation at the narrow passage near A23F and G575 blocks the migration of carbonate and results in less than 4% activity for the synthesis of carbonate phosphate [175].

In the case of multifunctional enzymes, substrate channeling results in faster multi-step catalysis, because substrates do not equilibrate with the environment exterior to the catalytic complex, resulting in higher local concentrations for subsequent steps in the process [163,164,176]. Experimental work has shown that catalytic assemblies within cells can use substrate access tunnels to channel substrate from one protein to another, greatly enhancing rates. For instance, structural studies of hen ovotransferrin have indicated an anion directing track [177]. Furthermore, kinetics studies of the bifunctional *Escherichia coli* enzyme proline utilization A (PutA), which catalyzes the oxidation of L-proline to L-glutamate in two successive reactions, exhibits substrate channeling between the proline dehydrogenase (PRODH) and Δ^1^-pyrroline-5-carboxylate dehydrogenase (P5CDH) components of PutA [178]. In computational studies, MD simulations were used to investigate substrate channeling in DmpFG bifunctional enzymes from aldolase (DmpG) to dehydrogenase (DmpF) subunits using a series of progressively larger substrates [179]. Water molecules were found to specify a route for aldehyde products toward the channel. The size of the aldehyde substrate influenced the free energy changes associated with substrate channeling and entrance into the dehydrogenase active site. For channeling from the first active site to the second active site, the overall change in free energy was −3.3 kcal/mol for acetaldehyde and −1.8 kcal/mol for butyraldehyde. Acetaldehyde was required to cross an energy barrier of 2.2 kcal/mol just before entering the dehydrogenase active site, while butyraldehyde encountered an energy barrier of 5.7 kcal/mol. Simulation results showed that the proper orientation of aldehyde products was requisite for entrance in the channel, which increases the entropic cost of channeling and slows down the process for the extended conformation of butyraldehyde. Characterization of substrate channels will be useful to tailor DmpFG by mutating channel lining residues. For instance, simulations have shown the in silico I159A mutant is able to transport the relatively bulky substrate benzaldehyde, which was found unlikely in wild type.

##### Substrate Gating

Substrate binding and tunneling is associated with small- to large-scale conformational rearrangements, which range from side chain rotations to loop motions, as mentioned in previous sections. These conformational changes and associated dynamics have been shown to be an intrinsic property of various proteins, which indicates that a gating mechanism exists in some proteins for controlled entrance/exit of substrate and solvent molecules to/from the protein interior [11,28,161,162]. The movement of protein regions involved in gating mechanisms gives rise to open and closed protein conformations and influences the solvation of cavities. Experimental and computational studies of lipases characterized the lid subdomain, which is located over the active site and controls substrate access to the enzyme active site by interfacial, temperature-switch, and aqueous activation [180,181]. Lid movement gives rise to open and closed conformations in the presence of a hydrophobic substrate and modulates activity, substrate specificity, and thermostability [180]. MD simulation of lipases in water and organic solvent indicates that active site accessibility to substrates is solvent-dependent. Namely, a lid-closed conformation is favored in water, while lid-opening is promoted in organic solvent [182,183]. Interfacial activation of lid opening was observed in MD simulations of both T1 lipase [183] and CALB [184,185] on hydrophobic surfaces. Blank et al. used the multistate Bennett acceptance ratio [186] (MBAR) method to estimate occupancies of different CALB conformations in solution [184]. Recent NMR studies of human serine hydrolase monoacylglycerol lipase (hMGL) reported the involvement of inter-residue aromatic interactions and H-bond networks to regulate open–closed conformational transitions [187]. Results demonstrate the involvement of global conformational changes along with lid-gating dynamics in the population of open–closed states. MD simulations of P450BM-3 monooxygenase in aqueous dimethylsulfoxide (DMSO) solution showed the closing of the substrate access channel by DMSO molecules, with a single mutation (F87A) that makes the active site accessible to the DMSO molecule [188–190].

##### Proton and Electron Transfer

During catalysis, proton transfer through the dynamic pathways of enzymes may be comprised of H-bond networks of amino acid residues and water molecules. The zinc bound metalloenzyme human carbonic anhydrase II (HCA II) has been studied extensively using structural, kinetics, mutagenesis, and simulation studies to gain mechanistic insight into its rate-determining proton transfer step [49,191,192]. HCA II-mediated catalysis involves the formation of bicarbonate as a result of a nucleophilic attack on carbon dioxide by a zinc-bound hydroxide, followed by intramolecular proton transfer from the zinc-bound water molecule to His64 through a hydrogen-bonded water cluster. In the active site, the protonation state of His64 influences its side chain orientation and modulates the distance between zinc and the imidazole ring, which is 8 Å and 10 Å in inward and outward conformers, respectively [193]. Proton transfer seems to be optimized by the conformational switching of His64 and the associated reorganization of water clusters between His64 and zinc-bound water. Transition path sampling studies showed rearrangement in the residues Asn62, Trp5, and Tyr7 during the conformational transition of His64 side chain [194]. Water molecules inside the active site cavity and the residues Asn62 and Asn67 were found to be coupled to His64 conformational dynamics. Multistate empirical valence bond (MS-EVB) simulations along with transition path sampling observed the coupling of protein dynamics and catalysis and showed involvement of several active site residues in a proton transfer event [195,196]. Clearly, the atomistic details provided by simulations deepen insight into small molecule participation in enzyme mechanisms.

An atomistic MD docking simulation of the P450BM-3 heme domain was performed to investigate preferential binding modes and electron transfer (ET) from an ET mediator, cobalt(II) sepulchrate (Co(II)Sep), to the heme iron in solution at different Co(II)Sep concentrations [138]. Results provided new insights into the ligand adsorption mechanism, through detailed binding site mapping on the protein surface. Protein conformational dynamics were significantly affected by the Co(II)Sep concentration, with reduced flexibility of the surface exposed loop regions due to Co(II)Sep binding. Co(II)Sep-bound protein complexes were used to estimate ET rates using different ET tunneling pathways from Co(II) to heme iron using the Pathways model [197,198]. Increased Co(II)Sep concentration was found to induce opening of the substrate access channel, which can affect the catalytic activity, as observed experimentally [199]. In the given protein conformation, the Pathways program identifies an effective ET coupling by evaluating the highest electronic tunneling coupling (*T_DA_*) through different pathways connecting the donor (Co(II)) and the acceptor (heme Fe) through bonds and space, and estimates the ET rate (k_ET_) [197,198]. Only a few binding sites provided efficient ET pathways to the heme iron, by yielding ET rates of *k_ET_* ≥ 10 s^−1^. Notably, higher rates were observed from binding regions close to the solvent access channel and the interface of the flavin mononucleotide (FMN) and heme cofactor binding domains. MD simulations of the FMN–heme complex observed seven ET pathways using the Pathways model [197,198], while three ET pathways had *k_ET_* values comparable to the experimental values [200]. The FMN–heme complex was observed to go through an interdomain structural rearrangement during simulations to reduce the distance between FMN and heme cofactors from 18.1 Å in the crystal structure to ~14.1 Å, which can provide a favorable ET rate comparable to the experimental *k_ET_* value of 80 s^−1^ [200,201]. Activation of different ET pathways was found to be affected by collective dynamics of the FMN–heme complex along the trajectory. Through a PCA-based approach, the first two essential modes were found to be correlated with the first two ET pathways having high *k_ET_* values, which indicates the role of protein dynamics in ET processes [202,203].

#### 2.2.2. Cofactor Binding

MD simulations have been used to gain an atomistic understanding of bond making/breaking and electron/proton transfer. Binding of non-substrate ligands is also critical in many enzymes for optimum catalytic activity. In some cases, enzymes attain specific states by modifications in their covalently bound cofactors, such as changes in protonation state. It has been observed previously that different protonation states of the cofactor induce conformational changes [204] and also influence the cofactor binding affinities [205] of a protein. MD simulations were used to investigate the effects of protonation states of FMN cofactors on the conformations and dynamics of the FMN-binding domain of P450BM-3 monooxygenase as holo- and apo-protein in solution [204]. The PCA calculations have shown significant differences in atomic fluctuation amplitudes in holo- and apo-protein simulations. The protonation states of the isoalloxazine ring influenced the H-bond network, resulting in conformational rearrangement in the binding site. The properties of the FMN-binding pocket, such as volume, hydrophobicity, solvent accessibility, and polarity were estimated using the MDpocket [206] program. MDpocket detects and characterizes the binding pocket in an ensemble of protein conformations using a grid based methodology [206]. Reduced FMN cofactor strengthens the H-bond network between FMN cofactor and protein; a major conformational change was observed in the orientation of Trp574 residue that is critical for cofactor binding and ET tunneling from FMN to heme. In the absence of cofactor, high fluctuations were observed in the FMN binding region, which can promote feasible rebinding of the FMN cofactor, as observed experimentally [207]. Recently, ultrafast fluorescence spectroscopy demonstrated vibrational coupling of FMN motion and protein to the FMN excited state in the flavoenzyme pentaerythritol tetranitrate reductase (PETNR) [208]. Low frequency vibrational modes, such as a butterfly bending motion, influenced transition state formation. Protein and FMN vibrations on nanosecond-to-femtosecond time scales were found to affect the rate of hydride transfer in the transition state. Along these lines, mutagenesis, modeling, and kinetic studies of propanediol oxidoreductase (FucO), an NADH-dependent enzyme from *Escherichia coli*, show enhancement in enzymatic activity by mutating cofactor binding site residue F254I, which increases the dissociation rate of the cofactor from the active site [209]. MD simulations of horse liver alcohol dehydrogenase (HLADH) as substrate-bound, intermediate, and product complex revealed the proton shuttling and rate-determining hydride transfer step [210]. Conformational changes in cofactor were shown to be critical toward transition state stabilization and selection of a hydrogen-bonded relay series of amino acid residues for proton transfer to water. In HLADH, three pathways were observed for dynamic proton shuttling to water from His51 or NAD^+^ ribose 3′OH, along with an accumulation of water at either of the two sites. Recently, cofactor-involved product release in DHFR was observed in NMR relaxation dispersion and stopped-flow kinetics experiments [211,212]. NMR studies demonstrate the involvement of protein dynamics in the product-release step in DHFR due to correspondence of the conformational fluctuations (12–18 s^−1^) in the active site of DHFR-product complex with the kinetics of product release (12.5 s^−1^) [212]. Reduced NADPH cofactor was found to affect the rate of product release through steric repulsion during conformational sampling of a state (the “closed excited state”). There is some variation in conformational dynamics (rates between exchange of states) when DHFR-product complex is bound to oxidized (NADP^+^) vs. reduced (NADPH) cofactor, but conformational exchange rates measured by NMR are of the same order of magnitude (1890 ± 80 s^−1^ bound to NADPH vs. 1420 ± 70 s^−1^ when bound to NADP^+^). In other work investigating the influence of cofactor on protein dynamics function, the effects of cofactor binding on protein folding mechanisms were investigated using small-molecule atomic force microscopy [213], spectroscopy [214], and MD simulations [215] combined with the Markov state model (MSM) [216,217]. MSM, a kinetic model, is constructed from detailed atomic MD simulations, and can provide relevant timescales, statistical significance and coarse-grained representations of the process under study [218]. Additionally, computational modeling and simulation of *Escherichia coli* thiol-disulfide oxidoreductase (TrX) demonstrated the involvement of an inter-domain twisting motion for synchronized cofactor capture and release during substrate binding and redox activity [219].

#### 2.2.3. Allosteric Sites

Protein function can be modulated through allosteric effects during catalysis, where effector binding at a distal site changes the substrate affinity and catalytic efficiency at the active site of the protein [220]. Effector binding changes the free energy landscape of a protein’s conformational space, and modulates conformational dynamics and transitions. NMR spectroscopy and MD simulation studies have been applied successfully to investigate the role of conformational dynamics in protein allosteric regulation [221,222]. MSMs built on multiple independent atomistic MD simulations shed light on ligand binding mechanisms in and around the active site in lysine-, arginine-, and ornithine-binding (LAO) proteins [24]. In MSM analysis, ensembles from long simulations can be pooled and clustered into microstates based on criteria such as root mean square deviation. Across the trajectory ensembles, the statistics of transitions between microstates can be used to create a MSM to access the essential dynamics and kinetics of binding events. Ligand binding in LAO was identified as occurring by multistate pathways with three dominant states, namely ligand-free open, ligand-bound closed, and a partially closed encounter complex. Ligand-free protein is unable to access the closed state, but it can access the partially-closed encounter complex state, which indicates a conformational selection mechanism in LAO binding. The presence of a ligand in the binding site triggers an induced-fit mechanism for transitions from the encounter complex state to the closed state. In another example, simulations of a calcium-binding calmodulin (CaM) domain showed binding-induced folding at higher calcium concentrations, while combined folding and induced fit occurred at lower calcium concentrations [223]. MD simulations of a homodimer dihydrodipicolinate synthase (DHDPS) revealed the role of the solvent-exposed Arg140 residue in the stabilization of the catalytic triad and binding of pyruvate as a substrate [224]. DHDPS simulations identified several metastable intermediates with favorable pyruvate interaction sites, and analyzed pyruvate binding pathways and kinetics, with researchers using MSM. MD simulations of peptide-prolyl cis–trans isomerase (Pin1) identified allosteric pathways and suggested that the preorganization of the catalytic site is induced by substrate binding on its N-terminal WW domain, which involves closure of the loop regions surrounding the substrate-binding cleft [225]. Simulations were able to capture the conformational dynamics required for allosteric communication in Pin1 at timescales up to nanoseconds. Meanwhile, CypA was studied using microsecond MD simulations to demonstrate variations in residue–residue contact dynamics and communication across CypA upon substrate binding that ranges from a few picoseconds to hundreds of nanoseconds time scales [226]. Finally, the positions of conserved water molecules in caspase-3 protein were found to be correlated with the sites of posttranslational modifications, suggesting their integral role in allosteric mechanisms and conformational selection [227]. Altogether, these studies indicate that allosteric processes facilitate the transmission of intra-protein information over long distances, and spotlight the influence of small molecules in enzyme catalysis.

## 3. Concluding Remarks

In this review, the contributions of MD simulation studies towards understanding the influence of small molecule interactions on protein conformational dynamics have been briefly summarized. There is ample experimental evidence supporting the fact that interactions of small molecules, namely solvents, substrates, and cofactors, profoundly impact enzymatic function. Recent advances in experimental techniques are continually evolving our knowledge of small molecule effects in enzyme catalysis, and provide critical validation of computational simulation techniques and their results. In silico studies have already made significant contributions toward understanding small molecule participation in biomolecular processes, particularly by providing spatial and temporal resolution that complement spectroscopic measurements. A number of analysis methods strengthen the capabilities of simulation studies by facilitating the drawing of statistically-sound conclusions from biomolecular MD trajectories in an approachable time scale. These include cluster analysis for conformational changes, normal mode analysis (NMA) [228] and principal component analysis (PCA) [229] for dominant functional motions of proteins; free energy profiles (FEP) for ligand binding, activation and catalysis [5]; Markov state model (MSM) [230] for decomposition of conformational sampling, protein dynamics, and kinetics modeling; and transition-state free energy profiling to identify reaction mechanisms and compute transition rates [231,232]; Caver to identify tunnels and cavities in static and dynamics protein; and the Pathways path model [197,198] to identify effective electron transfer coupling (*T_DA_*) and rate (*k_ET_*). Additionally, MD simulations combined with quantum mechanics and molecular mechanics (QM/MM) [153,154] have been shown to provide high levels of accuracy in the calculation of energy profiles and barriers to obtain mechanistic information, reaction pathways, and reaction rates [154]. Efficient sampling of energy landscapes can be obtained by using advanced enhanced sampling techniques, namely replica exchange molecular dynamics, metadynamics and simulated annealing [5,233,234].

Careful methodology is required in protein simulations to get adequate sampling of the potential energy surface. Therefore, the long trajectories used for biological macromolecules allow for excellent statistical sampling of small molecule dynamics, such that values for uncertainties can and should be considered when investigating small molecule effects on enzyme catalysis. Analysis of small molecule interactions and dynamics can be carried out through calculation of diffusion; hydrogen-bond analysis and lifetimes; heat maps of probabilities of interaction; orientational analysis of substrates and reorientation times of solvents; and free energies through −RT*ln* P (P = probability) for affinity in substrate-access channels, linear interaction energy relationships [235–238], and MM/PBSA and MM/GBSA methods [239,240] for ligand binding affinities (molecular mechanics energies with Poisson–Boltzmann (PB) or generalized Born (GB) and surface area continuum solvation). A more comprehensive understanding of solvent effects, substrate affinities, and cofactor and allosteric effects on protein structure–dynamics–function is required for a holistic view of enzyme catalysis, and computational methods are poised to make significant contributions in this area.
